# Development of Diclofenac Sodium 3D Printed Cylindrical and Tubular-Shaped Tablets through Hot Melt Extrusion and Fused Deposition Modelling Techniques

**DOI:** 10.3390/ph16081062

**Published:** 2023-07-26

**Authors:** Tryfon Digkas, Alina Porfire, Jeroen Van Renterghem, Aseel Samaro, Gheorghe Borodi, Chris Vervaet, Andrea Gabriela Crișan, Sonia Iurian, Thomas De Beer, Ioan Tomuta

**Affiliations:** 1 Laboratory of Pharmaceutical Process Analytical Technology, Ghent University, Ottergemsesteenweg 460, 9000 Ghent, Belgium; tryfon.digkas@ugent.be (T.D.); jeroenvanrenterghem@hotmail.be (J.V.R.); thomas.debeer@ugent.be (T.D.B.); 2 Department of Pharmaceutical Technology and Biopharmacy, Faculty of Pharmacy, University of Medicine and Pharmacy “Iuliu Hațieganu”, 41 Victor Babeș Street, 400012 Cluj-Napoca, Romania; crisan.andrea@umfcluj.ro (A.G.C.); iuriansonia@yahoo.com (S.I.); tomutaioan@umfcluj.ro (I.T.); 3 Laboratory of Pharmaceutical Technology, Ghent University, Ottergemsesteenweg 460, 9000 Ghent, Belgium; aseelsamarou.as@gmail.com (A.S.); chris.vervaet@ugent.be (C.V.); 4 National Institute for Research and Development of Isotopic and Molecular Technologies, 65-103 Donath Street, 400293 Cluj-Napoca, Romania; g_borodi@yahoo.com

**Keywords:** three-dimensional printing (3DP), hot melt extrusion (HME), quality by design (QbD), release kinetics

## Abstract

The present study aimed to develop 3D printed dosage forms, using custom-made filaments loaded with diclofenac sodium (DS). The printed tablets were developed by implementing a quality by design (QbD) approach. Filaments with adequate FDM 3D printing characteristics were produced via hot melt extrusion (HME). Their formulation included DS as active substance, polyvinyl alcohol (PVA) as a polymer, different types of plasticisers (mannitol, erythritol, isomalt, maltodextrin and PEG) and superdisintegrants (crospovidone and croscarmellose sodium). The physicochemical and mechanical properties of the extruded filaments were investigated through differential scanning calorimetry (DSC), X-ray diffraction (XRD) and tensile measurements. In addition, cylindrical-shaped and tubular-shaped 3D dosage forms were printed, and their dissolution behaviour was assessed via various drug release kinetic models. DSC and XRD results demonstrated the amorphous dispersion of DS into the polymeric filaments. Moreover, the 3D printed tablets, regardless of their composition, exhibited a DS release of nearly 90% after 45 min at pH 6.8, while their release behaviour was effectively described by the Korsmeyer–Peppas model. Notably, the novel tube design, which was anticipated to increase the drug release rate, proved the opposite based on the *in vitro* dissolution study results. Additionally, the use of crospovidone increased DS release rate, whereas croscarmellose sodium decreased it.

## 1. Introduction

Nowadays, three-dimensional printing (3DP) technology is promoting innovations in the pharmaceutical industry by enabling the production of custom-made personalised medicines. The concept of personalised medicine aims to provide patients with treatments adjusted to their individual needs [1]. That can be accomplished by combining a patient’s pharmacogenomics with information related to their diet, lifestyle and pathophysiology. So far, global pharmaceutical companies have mainly manufactured commercially available pharmaceutical dosage forms with universal predetermined drug doses, using processes which require high production costs, large industrial footprint, experienced personnel and a variety of time-consuming batch-manufacturing steps. On the other hand, 3D printing technology offers the opportunity to reproducibly generate a wide range of pharmaceutical dosages in a short manufacturing time, tailored to meet the unique healthcare characteristics, needs, and preferences of individual patients. Furthermore, by precisely manipulating the spatial distribution of multiple active pharmaceutical ingredients (APIs) within a single dosage form, control over drug release kinetics can be easily achieved [2,3]. Such customisation is feasible using 3D printing technology, even for dosages with complex geometries that would be challenging to produce using traditional power compression methods. Currently, there are various 3D printing techniques under investigation, such as fused deposition modelling (FDM), powder bed printing, binder jetting, selective laser sintering and stereolithography, but FDM stands out as one that offers the most immediate potential for personalising dosage forms to suit patient’s requirements [4].

FDM is an extrusion-based 3D printing technique that allows manufacture of drug-loaded dosage forms by simultaneously melting, extruding and depositing thermoplastic filaments layer-by-layer in a ‘writing’ mode. FDM is a widely investigated method for the manufacturing of pharmaceutical oral dosage forms due to its ease of application, flexibility in producing versatile dosage form designs and cost-effectiveness. One of the advantages associated with the FDM method is that it allows the fabrication of personalised dosage forms with tailored dosage strengths and drug release profiles at a relatively low production cost by utilising different drug-carrier polymers (i.e., insoluble, immediately soluble, enteric soluble and swellable/erodible biodegradable polymers) or simply by modifying the size, shape, geometry and density of the printed dosage forms [5,6]. Thus, FDM is an easily accessible, low-cost, versatile printing technique, highly advantageous for manufacturing personalised medicines [7,8]. However, the nature of the FDM printing technique leads to three significant challenges for its application to produce personalised solid dosage forms. Firstly, a hot melt extrusion (HME) process is required as an additional upstream processing step to produce custom-made filaments used as FDM loading material. In this regard, HME is a widely used processing technology allowing the molecular dispersion of active ingredients within polymer matrices in the form of an extruded filament. Therefore, HME can be used to improve the solubility and bioavailability of poorly water-soluble active pharmaceutical ingredients as well as for developing systems with sustained, modified and targeted drug delivery [6]. Secondly, the fused deposited material requires a low melt viscosity at the printing temperature, suggesting the need for using highly thermoplastic materials, which most of the pharmaceutical grade polymers are not [9]. Thirdly, the FDM filaments require distinctive mechanical and rheological properties to accurately build up a 3D printed object. However, the aforementioned barriers can be overcome by selecting appropriate polymers. In most reported cases, the 3D printing of oral solids has been performed using polylactic acid (PLA) [10], polyvinyl alcohol (PVA) [5,11,12,13] or poly-acrylics (Eudragit) [14].

Diclofenac sodium (DS) is a non-steroidal anti-inflammatory drug (NSAID) with analgesic, anti-inflammatory and antipyretic properties. Currently, DS is commercially available in oral, intravenous, suppository, transdermal patch or gel formulations and is one of the most prescribed NSAIDs for topical or systemic use to reduce inflammation and pain associated with osteoarthritis, rheumatoid arthritis and ankylosing spondylitis [15]. However, it is important to note that oral diclofenac treatment is associated with major side effects, such as cardiovascular, gastrointestinal and hepatic complications [15]. Given these concerns, DS emerges as an ideal candidate for the fabrication of FDM 3D printed tablets with personalised dosages, which can particularly be adjusted for elderly patients with concomitant hepatic dysfunction or cardiovascular problems. Furthermore, the high stability of DS under high processing temperatures [16] makes it a suitable model drug for producing drug-loaded polymeric filaments through HME processing [17]. Another important aspect of DS is its classification as a BCS type II drug, which is characterised by poor solubility and high permeability properties. Additionally, its physical–chemical stability is pH dependent, as it is insoluble in the gastric fluid conditions (pH 1.2) but highly soluble in intestinal fluids (pH 6.8) and water [18].

PVA is one of the most widely investigated polymeric matrices in FDM 3D printing due to its non-toxic, biodegradable, swellable, thermoplastic and water-soluble properties [1]. Its melting point may range from 180 °C (partially hydrolysed) to 228 °C (fully hydrolysed), depending on the degree of hydrolysis of the acetate groups. These aspects make PVA a well-suited polymer for HME. Currently, an approach employed to enhance drugs’ physical stability and improve their dissolution and oral bioavailability is the utilisation of amorphous solid dispersions (ASDs). These systems involve combining the API within a polymeric carrier to form a homogenous and amorphous binary mixture, offering long-term physical stability of the amorphous API. Considering the above aspects, the objective of this study was to develop DS-loaded 3D printed dosage forms, using custom-made PVA filaments. To achieve this, DS was chosen as model drug with poor aqueous solubility, and the HME process was employed to enhance the drug’s dissolution rate through its ASD within the water-soluble polymer. In addition, different types and concentrations of plasticisers were investigated to determine their impact on the mechanical properties of the custom-made PVA filaments loaded with DS. The overall study was accomplished through a quality by design (QbD) approach and by implementing quality risk management (QRM) tools combined with an experimental statistical design.

## 2. Results and Discussion

The development of 3D printed tablets is a complex procedure involving formulation design (active ingredients and excipients) and processability of materials (miscibility of the components, extrudability and printability), so as to ensure consistency of the manufacturing stages and reproducibility of the results. Having this in mind, 3D printed tablets were developed through a combination of HME and FDM 3D printing processes, where formulation and process variables which may affect the quality attributes of both intermediate (drug-loaded filaments) and final drug products (3D printed tablets) were evaluated in an attempt to design a product meeting the established quality target product profile (QTPP).

### 2.1. Preliminary Formulation Experiments

Preliminary studies were conducted to establish the qualitative and quantitative composition of the blends used for HME and to produce filaments with the adequate mechanical properties for FDM printing. Furthermore, these studies were carried out to identify the suitable process conditions for the formulations processed though HME. For this to be accomplished, pure PVA and DS-PVA blends were first extruded, in various temperature settings, as shown in Table 1. Although the drug–polymer mixture showed good miscibility, the extruded filaments were brittle, without plastic deformation, and thus displayed inadequate printing performance. Therefore, to improve the mechanical properties of the filaments, different types of plasticisers were included. The selection of plasticisers was based on previous studies showing that the most effective plasticisers are those that structurally resemble the polymeric matrix they are intended to plasticise [19]. Considering this, plasticisers with polyol functional groups, such as mannitol (MAN), erythritol (ERY) and isomalt (IM), were tested. In addition, the effectiveness of PEG 1500 and PEG 4000 as molecular weight forms of polyethylene glycol (PEG), as well as poloxamer 188 (Lutrol F68) as a polymer, was investigated.

The results obtained from the preliminary trials evidenced that extremely friable filaments were obtained using PEG4000, PEG1500 and Lutrol 68. That might be attributed to the high processing temperature settings used during HME compared with the low melting point of PEG1500, PEG4000 and Lutrol 68 at 46, 54 and 55 °C, respectively [20]. Among the different plasticisers investigated, filaments containing MAN, ERY and IM extruded under specific processing conditions (i.e., at 25, 160 and 180 °C for extruder zones 2, 3 and 4–7, respectively) exhibited superior mechanical properties, resulting in smooth feeding during FDM. Additionally, these findings indicated that the inclusion of crospovidone (PVPP) as a superdisintegrant did not negatively impact the mechanical properties of these filaments, indicating its good compatibility with the rest of the excipients processed via HME. Based on these results, further experimental studies were carried out using only MAN and ERY as plasticisers to systematically investigate their effectiveness at a concentration of 10 and 15% *w*/*w*.

### 2.2. QTPP of 3D Printed Tablets

As a first step of the QbD concept, the desired QTPP of the 3D printed tablets was defined. The critical quality attributes (CQAs), which represent the quality characteristics with the most critical impact on the desired product performance, were further determined. The QTPP and the derived CQAs were established based on scientific, regulatory and practical considerations and limitations. A summary of the desired QTPP is presented in Table 2, which was designed with the aim to produce DS-loaded FDM 3D printed tablets with a modified drug release profile in phosphate buffer of pH 6.8 [21].

### 2.3. Risk Identification and Evaluation

**Figure 1:**
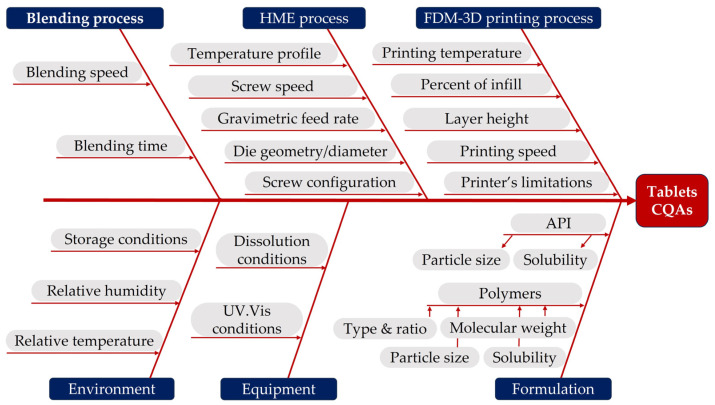
Diagram illustrating the factors that might impact on a 3D printed tablet’s CQAs.

A quality risk management approach involving risk identification and risk evaluation was implemented to identify, manage and control all the potential variables that could affect the predefined critical quality attributes. In this regard, the factors that could potentially affect the release kinetics from the 3D printed tablets were identified using the Ishikawa diagram (Figure 1).

Considering the increased number of identified factors, the failure mode effect analysis (FMEA) method was subsequently applied to examine the extent of failure of each identified factor and thereby minimise the number of variables that require further investigation through a screening DoE. The results of this analysis are summarised in Supplementary Table S1 and thoroughly discussed in the following paragraphs.

Previous studies have shown that the efficacy of FDM printing is dependent on the mechanical and rheological properties of the filaments employed [8,22,23]. Therefore, it is imperative to devise an initial formulation scheme that would enable obtaining drug-loaded filaments with the required properties for FDM application and ensure manufacturing 3D printed dosage forms conform to the desired QTPP. Considering this, the physical attributes of DS and the type and concentration of excipients were considered as critical formulation factors.

In regard to the HME process step, the extrusion temperature, screw speed, feed rate and the die specifications are significant parameters that have been previously investigated for their impact on filaments’ mechanical properties [24,25]. An improper control of these parameters may lead to poor printing performance of drug-loaded filaments or affect the dissolution behaviour of the printed tablets due to issues such as incongruent melting, incomplete dissolution of active ingredient in the polymeric matrix or thermal decomposition of the processed ingredients. Specifically, extruding at temperatures below an optimal range can cause incongruent melting or improper amorphization of the included active ingredient, which can significantly affect the dissolution performance of the printed tablets during downstream processing. In contrast, by extruding at higher than the optimal temperature settings, thermal decomposition of the processed ingredients can possibly occur [24]. Considering these, the optimal temperature settings were primarily identified and employed for the extrusion of eight DS-loaded filaments with varied types of superdisintegrant as well as types and concentrations of plasticiser. Thus, the chosen temperature settings were set above the glass transition temperature (Tg) of the extruded blends to ensure the miscibility of the melts and the effective dissolution of the drug within the polymeric matrices without decomposing the included components. The stability of the API within the extruded filaments was subsequently confirmed through thermal characterisation studies, which were conducted as a risk mitigation step prior to FDM printing. Alongside the extrusion temperature, screw speed and feed rate were identified as significant factors affecting the quality of the DS-loaded filaments. This is due to their direct impact on the mean residence time of the material during extrusion. Operating at high screw speeds can lead to insufficient exposure of the material to heat, leading to incongruent melting and incomplete dissolution of DS in the molten matrix. On the contrary, low screw speeds can lead to prolonged residence times and extensive exposure of the material to heat, which may result in the degradation of the included components. In addition, low shear rates can potentially increase compounds’ melt viscosity, which can lead to high torque values and extrusion blockage [24]. Therefore, the adequate process conditions related to the extrusion temperature, screw speed, feed rate and die geometry were identified and standardised during preliminary studies.

According to existing literature, printing conditions should be optimised in accordance with the rheological and mechanical properties of the filaments used [23]. Failure to do so may result in printing complications such as clogged nozzles, filament degradation, shrinking, warping, bend-breaking or inadequate adhesion of the deposited layers, which can result in printing failure [23]. Specifically, operating at printing temperatures exceeding a certain critical range may cause filament degradation during the feeding step of the process. Conversely, printing below the transient melting temperature of the filaments may result in improper flowability, a rapid solid–fluid–solid state transition and consequently an improper adhesion of the deposited layers. In addition to temperature, printing speed is another critical factor that must be carefully controlled. A high printing speed can lead to an improper solidification and adhesion of the deposited layers while a low operational speed can result in extrusion issues and tablet weight variabilities [26,27]. Considering the above aspects, in this study, although rheological characterisation of the extruded filaments was not performed, the adequate printing settings associated with temperature and speed were defined through preliminary trials.

Besides the previously discussed parameters, layer thickness was also defined as a critical factor since it can directly affect the surface area and porosity of the printed geometry, which, in turn, can influence the diffusion and dissolution rates of the API [27]. In fact, while a lower layer thickness can improve the deposited accuracy and tensile strength of the geometry, it may also increase the manufacturing time. On the contrary, a higher layer thickness can increase the number of pores on the surface, leading to higher wettability and improved dissolution rates [27]. Considering these aspects, this factor was investigated further.

In addition to the formulation factors, the importance of the design and dimensions of a printed dosage form in relation to the release profile of the loaded API has been widely reported in the literature [2,5,12,28]. In fact, the customisation of printed dosage forms’ design has emerged as a promising tool for tailoring drug delivery to meet specific therapeutic requirements [1]. In light of this evidence, the present study suggested the development of two custom-designed structures, such as a conventional cylindrical tablet design and an alternative tubular-shaped geometry, to fabricate DS printed dosage forms with the objective to enhance the drug’s release efficiency. The rationale behind our design selection was based on the premise that an increase in the surface area to volume ratio can potentially enhance the dissolution rate of the drug from the printed dosage forms, as previously reported [5,17,29].

Considering these issues, FMEA highlighted five factors as potentially critical for the overall DS release performance from the printed dosage forms. These were, namely, the type of superdisintegrant, type of plasticiser, plasticiser concentration, thickness of the printed layer and tablet’s geometry. According to the FMEA results as presented in Supplementary Table S1, these factors evidenced the highest RPN values and therefore were subsequently considered for further investigation through a screening experimental study.

### 2.4. Filaments’ Preparation and Characterisation

**Figure 2:**
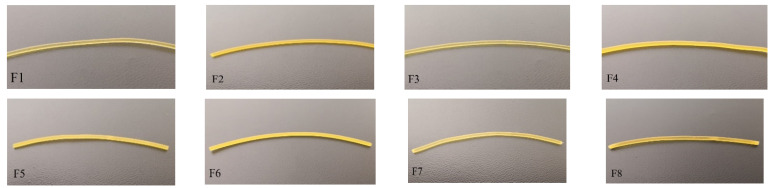
Hot melt extruded filaments F1–F8.

Risk analysis showed that both the diameter and the mechanical properties of the custom-made filaments are critical parameters for a successful FDM process. To achieve extruding filaments with the desired shape and diameter, the twin-screw extruder was equipped with a cylindrical steel die of 1.80 mm. The extruded filaments were then pulled away from the die with a custom-made pull-roller system, regulated at a certain speed to obtain filaments with a 1.75 mm diameter. Based on these actions, the average diameter of the obtained filaments was 1.70 mm with observed variations of ±0.10 mm occurring every 0.5 m in length. Moreover, as depicted in Figure 2, all the obtained extrudates exhibited a noticeable yellow discolouration, potentially attributed to the thermal degradation of sugar alcohols present in the formulation, such as MAN and ERY [30,31]. Additionally, all the extruded filaments were transparent, indicating the ASDs of diclofenac sodium within the polymer matrix. With respect to the loss on drying measurements, the findings evidenced that moisture content was absorbed from all the filaments after the HME process (Table 3). That may be attributed to the highly hygroscopic nature of PVA in combination with its high concentration (67–72%) within the filaments. Notably, formulations containing higher concentrations of plasticiser (F3, F4, F5 and F6) demonstrated an increase in their moisture content. This observation can be ascribed to the combined effect of both the hydrophilic plasticiser and the polymer molecules, which can synergistically contribute to enhancing the capacity for water absorption by the polymer matrix [32].

In regard to the processing of filaments through HME, an important parameter that contributed to characterising the energy consumption of the extruder’s motor was the torque value (Nm). Keeping the processing parameters such as temperature, screw speed and feed rate constant throughout the extrusion process, the energy consumption was mainly affected by the type and concentration of the included plasticisers. In this context, extrudates containing 15% *w*/*w* plasticiser (F3, F4, F7 and F8) displayed lower torque and SME values compared to those containing 10% *w*/*w* plasticiser (Table 3). That could be explained by the fact that a higher concentration of plasticiser can decrease the melt viscosity of the formulation inside the screw channel, resulting in lower screw stress and thus reduced torque and SME values. Moreover, the type of plasticiser had no significant impact on the torque and mechanical energy. However, filaments with PVPP displayed a better processability than those with CSSNa, since a lower amount of shear stress and therefore SME was observed in formulations including PVPP as a superdisintegrant (F1, F3, F5 and F7).

To achieve smooth feedability and precise deposition during FDM printing, filaments should possess adequate mechanical properties such as stiffness and strength. Brittle filaments will possibly be broken by the feeding gears during the loading phase since they break just after their elastic limit without significant plastic deformation, whereas filaments with high ductility will possibly deform and coil up around the drive gear. Therefore, to produce filaments with proper mechanical characteristics, formulation experiments were conducted by including mannitol and erythritol as plasticisers, while their plasticisation effect was investigated by adding them at 10% and 15% *w*/*w* concentration, respectively. Considering this, tensile measurements were applied to investigate the deformation and mechanical properties of the filaments under tension.

**Figure 3:**
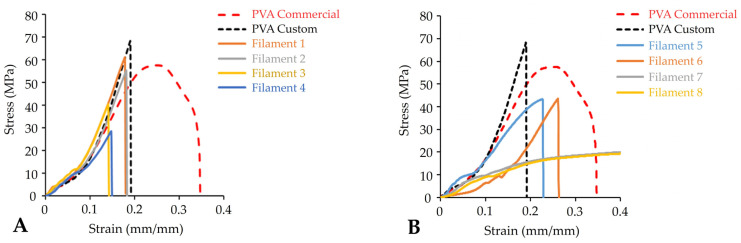
Tensile measurements of commercial and custom-made PVA filaments, filaments F1 to F4 (**A**); F5 to F8 (**B**).

The results evidenced that the commercial PVA filaments exhibited adequate feeding performance owing to their sufficient strength, ductility and toughness (Figure 3A). Conversely, custom-made pure PVA filaments showed low strain and the absence of plastic deformation, as fracture occurred immediately after the end of their elastic region, indicative of a brittle material with low ductility and energy absorption capacity (Figure 3A), which could not successfully be fed into the FDM printer. Filaments (F1 and F2) with low MAN content (10% *w*/*w*) had a similar mechanical profile to the PVA filaments prepared in this study (Figure 3A). On the other hand, the addition of 10% *w*/*w* ERY (F5 and F6) slightly improved the ductility of the filaments, whereas the strength and toughness were diminished (Table 3). By increasing the concentration of the plasticiser to 15% *w*/*w*, filaments containing MAN (F3 and F4) became weaker and less ductile as they withstood less stress and strain. Conversely, with the addition of ERY as plasticiser at 15% *w*/*w* (F7 and F8), the brittleness of the filaments was reduced with a significantly higher strain-bearing capacity (Table 3). Those filaments displayed a small plastic region with low strength (high breaking stress), high ductility (high breaking strain) and increased ability to absorb energy before fracture (Table 3), confirming their adequate feeding and printing performance. Despite the fact that printing difficulties occurred when using filaments with low plasticiser concentrations, tablets were successfully printed using all other filaments except those containing 10% *w*/*w* mannitol and PVPP (F2) due to persistent issues such as incomplete structure and surface gaps. Therefore, among the types and concentrations of plasticisers investigated, ERY at concentration of 15% *w*/*w* was considered the most suitable due to its superior plasticising effect, which resulted in filaments with improved mechanical properties.

**Figure 4:**
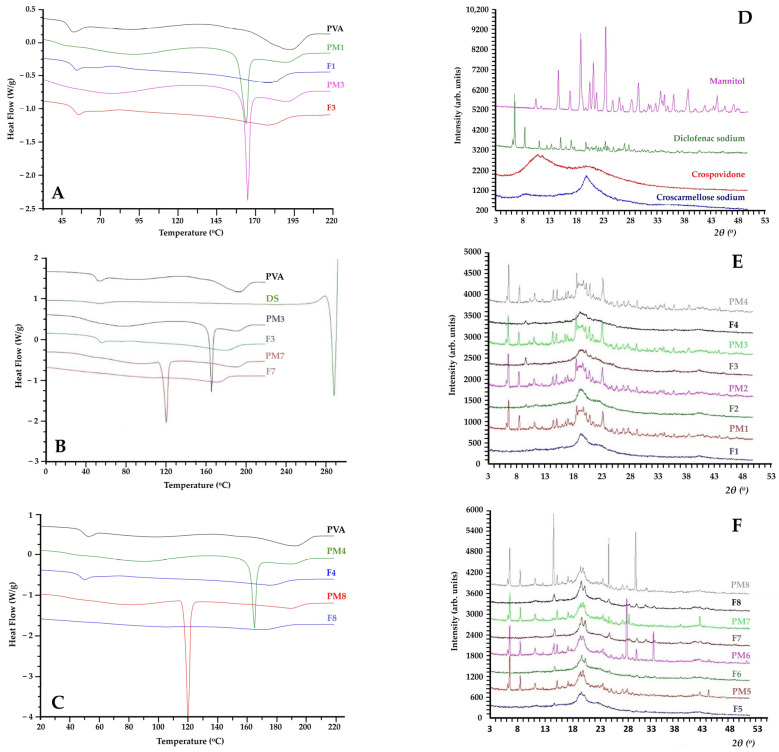
DSC thermograms of the first heating cycle for pure PVA and filaments (F)/physical mixtures (PM) 1–3 (**A**), 3–7 (**B**), 4–8 (**C**); X-ray diffractograms of pure DS, MAN, CSSNa and PVPP (**D**), filaments (F) and physical mixtures (PM) 1 to 4 (**E**) and 5 to 8 (**F**).

To assess the feasibility of producing homogenous and amorphous blends, differential scanning calorimetry (DSC) and X-ray powder diffraction (XRD) studies were conducted. The results of the thermal analysis of filaments are presented as DSC thermograms in Figure 4. As displayed in Figure 4A, samples of pure PVA exhibit three endothermic curves: the first between 50 and 70 °C, attributed to the polymer’s transition from glass to the elastic state, reaching a peak at 60 °C; a slight broad curve within the temperature range of 70–140 °C, associated with the gradual evaporation of water bound to the polymer matrix; and a third at 191 °C, indicating PVA’s melting point [20].

Physical mixtures 1 and 3 displayed a distinct and sharp endothermic peak at 165 °C, which is indicative of the melting point of MAN, while the onset of its melting process was observed at 162 °C [33]. In contrast, filaments 1 and 3 exhibited two broader peaks at 185 and 180 °C, respectively, which align with the melting point of PVA present within the corresponding formulations. These results indicate that a lowered melting temperature was achieved for PVA within these filaments, as compared to both their corresponding physical mixtures and pure PVA (Figure 4A). This can be ascribed to the presence of different concentrations of the plasticiser (MAN) in the polymer matrix. In addition, the DSC thermograms presented in Figure 4B revealed that physical mixtures 3 and 7 corresponding to PVA-DS-CSSNa-MAN and PVA-DS-CSSNa-ERY formulations, respectively, exhibited two sharp endothermic peaks at 165 °C and 120 °C. These peaks correspond to the melting temperatures of MAN and ERY, respectively. Notably, the absence of melting events observed in the thermograms of their corresponding filaments (F3 and F7) confirms the amorphous dispersion of the plasticisers within the polymer matrix, thus indicating their compatibility with PVA (Figure 4B). Additionally, the melting temperature of the PVA in the filaments was reduced to 180 °C and 172 °C for those plasticised with MAN and ERY, respectively. Figure 4C demonstrates similar thermal patterns for filaments 4 and 8.

For DS, DSC thermograms revealed a small endothermic peak at 50 °C, corresponding to water evaporation [34], followed by a sharp melting peak at 285 °C, indicating that the DSC analysis was unable to be conducted without observing degradation of the included excipients [16]. Therefore, to confirm the obtained DSC results and validate the incorporation of the initially crystalline DS as an amorphous dispersion within the PVA matrix, all the developed physical mixtures and their solid dispersions were analysed using the XRD method.

The findings of the XRD analysis performed on the raw materials, physical mixtures, and hot melt extruded filaments are displayed in Figure 4D–F. The X-ray diffractograms presented in Figure 4D revealed multiple distinctive peaks indicating the crystalline nature of pure DS, MAN, PVPP and CSSNa. The crystalline nature of pure DS was evidenced from the presence of multiple distinctive peaks appearing at 6.7, 8.5, 11.2, 15.1, 19.9, 23.5, 25.1, 27.1 and 27.9° 2θ, a result which is in accordance with other studies [28,35]. Similarly, distinctive diffraction peaks were detected for pure MAN, PVPP and CSSNa. By comparing the X-ray diffractograms of the physical mixtures (PM1–PM8) and their corresponding filaments (F1–F8), it is evident that the peaks disappeared while the intensity of some peaks was significantly reduced (Figure 4E,F). These results confirm the amorphous dispersion of DS and the involved excipients within the PVA-based matrices. These findings are in agreement with the results derived from the DSC analysis, confirming that the melt processing method and the chosen extrusion conditions were suitable for the amorphisation and solubilisation of the drug within the used polymeric matrices.

### 2.5. 3D Printed Tablets

**Figure 5:**
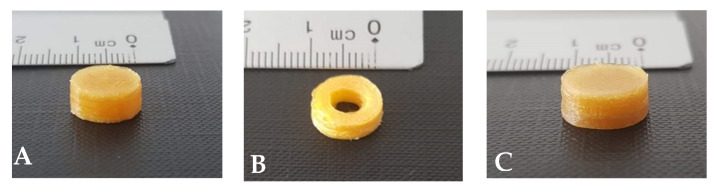

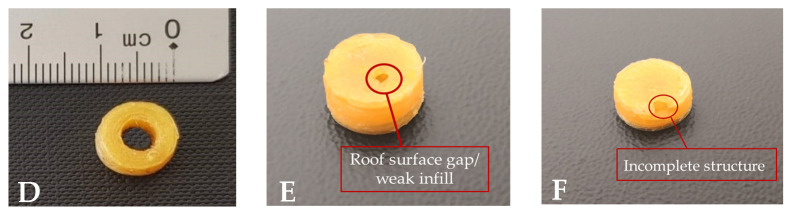
The 3D printed cylindrical-shaped tablet with 0.2 mm LH (**A**), tubular-shaped tablet with 0.2 mm LH (**B**), cylindrical-shaped tablet with 0.05 mm LH (**C**) and tubular-shaped tablet with 0.05 mm LH (**D**); 3D printed tablet with weak infill and gaps (**E**) and 3D printed tablet with weak infill and incomplete structure (**F**).

Recent studies have prove that both the geometrical shape and the surface area of 3D printed tablets may modulate the drug dissolution profile [2,5,28]. Taking this into consideration, two different 3D printing geometries (cylindrical and tubular) were investigated in this study to evaluate their impact on the drug release kinetics. Both shapes were designed with the same ratio of length, width and height, resulting in tubular-shaped tablets (Figure 5B,D) with lower drug loading and volume but a larger surface area compared to cylindrical tablets (Figure 5A,C).

The mechanical properties of all the printed tablets were satisfactory, exhibiting ease of handling and lack of friability. These observations align with several previous studies reporting zero friability and excellent resistance to breakage during handling for 3D printed tablets [2,29,36]. It has also been reported that printed tablets can maintain their shape, form and structure, remaining mechanically resistant even with design variations or channels [29]. In addition, traditional tablet hardness testers are often unsuitable for accurately assessing the hardness of printed tablets due to their high mechanical resistance to breakage [23]. Considering these, the developed DS printed tablets were not evaluated through friability and hardness tests.

To successfully print drug-loaded dosage forms, the risks involved in all the FDM input variables were minimised and controlled. As was previously described in Section 2.3, printing temperature and speed are high-risk FDM parameters that could impact the printing performance and hence the quality attributes of the printed tablets. Therefore, adjusting the filament’s heating and cooling temperatures was prerequisite to ensure material’s continuous flow from the heating chamber of the printing nozzle while ensuring rapid solidification and adequate adhesion to the building plate. In this regard, a printing temperature in the range between 160 and 170 °C was considered too low since nozzle clogging and poor adhesion to the building plate were observed, while at printing temperatures exceeding 195 °C brown colouration of the printed material was observed, indicating degradation caused by overheating. Based on preliminary trials, the adequate printing temperature was identified as 185 °C, which was 5 °C above the extrusion temperature (zones 4–7), while the platform (building plate) temperature was set at 25 ± 3 °C.

Printing speed is another high-risk FDM variable that can impact the printing performance and the quality of the printed tablets. The default printing speed was considered too high since the produced tablets had an incomplete structure with gaps or incomplete infill (Figure 5E,F). That can be linked to the limited time for the printed material to cool and properly adhere to the previously deposited layers. However, these problems were avoided by reducing the default printing speeds of the ‘first layer’ and ‘floor surface fill’ from 30 mm/s and 90 mm/s to 20 mm/s and 40 mm/s, respectively, and for the ‘roof surface fills’ speed from 90 mm/s to 30 mm/s, which improved layer adhesion and thus yielded a printed tablet with a proper structure (Figure 5A).

Layer height (LH) is an FDM process variable that impacts on tablet’s printing resolution, accuracy and detail, ultimately affecting the quality of the printed tablets. While a lower LH provided a more accurate and smoother curved surface of the printed tablets, it also resulted in a longer printing time and higher costs as a larger number of layers are required to complete the structure. The default LH setting was 0.2 mm, representing the standard printing resolution, while a lower LH will result in a higher printing resolution. By varying the LH, the tablet mass and printing time were affected. Using a higher printing resolution (i.e., an LH of 0.05 mm), the tablet mass for both geometries (cylindrical and tubular) increased. Additionally, the improvement of the printing resolution resulted in a considerable extension of the production time for each 3D printed tablet of the DoE. Specifically, reducing the LH from 0.2 mm to 0.05 mm, the production time significantly increased from 3.3 to 12 min, respectively.

All formulations exhibited low mass variability, with mass variation coefficient being below 0.2%. These results indicated good uniformity in tablet mass, which aligns with the specified pharmacopeia limits [37]. However, noteworthy variabilities were observed between the developed printed tablets of the DoE, as shown in Table 4. Variations in mass were observed between tablets with tubular and cylindrical geometry. These results can be attributed to the differences in the internal available space of each geometry. Previous reports have highlighted that printed tablets with tubular and cylindrical designs, despite having the same external layers and volume, can significantly differ in their internal filling capacity [27]. In cylindrical tablets, the inner space constitutes the majority of the built structure, while in the tubular format, it represents only a small part of the object. Consequently, tubular-shaped printed tablets may have a mass up to 49% greater than cylindrical printed tablets [27]. Additionally, differences in mass were seen among tablets with the same geometry and layer height. This can be attributed to the different flowability potential of the produced DS filaments with varied concentration and type of the plasticiser [23]. Moreover, it has been reported that polymeric matrices are prone to water absorption and therefore a mass variability between the produced printed tablets may be seen, driven by the hygroscopic nature of the formulations [23].

### 2.6. DoE Analysis

For the development of 3D printed tablets, a full factorial experimental design with 32 experiments was conducted. After the completion of the DoE, the experimental data were fitted using a partial least square (PLS) model and the ANOVA parameters were calculated. An overview of the experimental results of the DoE is presented in Table 5. All the formulations prepared according to the experimental design were completely dissolved within 4 h in pH 6.8 dissolution medium (Table 5). However, the formulations with 10% MAN and CSSNa, for both the cylindrical and tubular geometry (N2, N4), failed to be printed at high resolution into a well-structured 3D design, leading to missing data in the response matrix.

Statistical analysis evidenced that all the responses were well fitted and predicted by the model as the regression coefficients had values equal to and above 0.7. 𝑄^2^ values were higher than 0.4 and the small differences between 𝑅^2^ and 𝑄^2^ (<0.3) for most of the responses indicated a good statistical model with good predictive power. Moreover, model validity was good for each response (>0.75).

Furthermore, two F-tests were performed through analysis of variance in order to evaluate the significance of the statistical models and their lack of fit. The significance of the models was tested by comparing the variances of the regression models with the residual and their lack of fit by correlating the model error with the replicate error. The first ANOVA results revealed highly significant models for all the responses, while the second results showed no lack of fit for the developed models as *p* values (for lack of fit) were > 0.05.

The DS release profile from the developed 3D printed tablets was investigated by means of *in vitro* dissolution studies. These tests were conducted to evaluate the suitability of the tablets’ geometry, the combination of the polymers included and the effect of LH on DS release profile in phosphate buffer media at pH 6.8. The dissolution profiles of the 3D printed tablets with different geometries, layer heights and compositions are presented in Figure S1 in the Supplementary Materials. The dissolution results evidenced that among the developed printed tablets, N28 and N31 achieved the fastest drug release, reaching nearly 90% within 45 min of dissolution. Notably, even though both tablets were composed of 15% erythritol and had tubular geometry, the type of superdisintegrant and layer height differed. This highlights that combining dissolution-promoting approaches, such as geometry modulation and the incorporation of a superdisintegrant, can be an effective method for achieving a rapid release of DS, regardless of the superdisintegrant type and LH used. In addition, the slower drug release was seen in tablets N8 and N20 with almost 89 ± 1.3% and 85 ± 3.8% achieved in 120 min of dissolution, respectively. Interestingly, these formulations were manufactured with low LH, low concentration of plasticiser and tubular design, whereas the type of superdisintegrant and plasticiser varied. These findings suggest that the addition of superdisintegrants as dissolution enhancers is not effective under certain conditions. Previous studies have also reported that CSSNa and PVPP can act as dissolution enhancers in 3D printing dosages to some extent, since the molten polymers’ disintegration properties and performance could be disturbed during thermal processing through HME and FDM printing [2]. It is important to consider that the reduction in LH from 0.2 mm to 0.05 mm resulted in an increased production time (from 3.3 to 12 min), where the thermal processing of the filaments during FDM printing may have impacted the disintegration mechanisms of CSSNa and PVPP.

**Figure 6:**
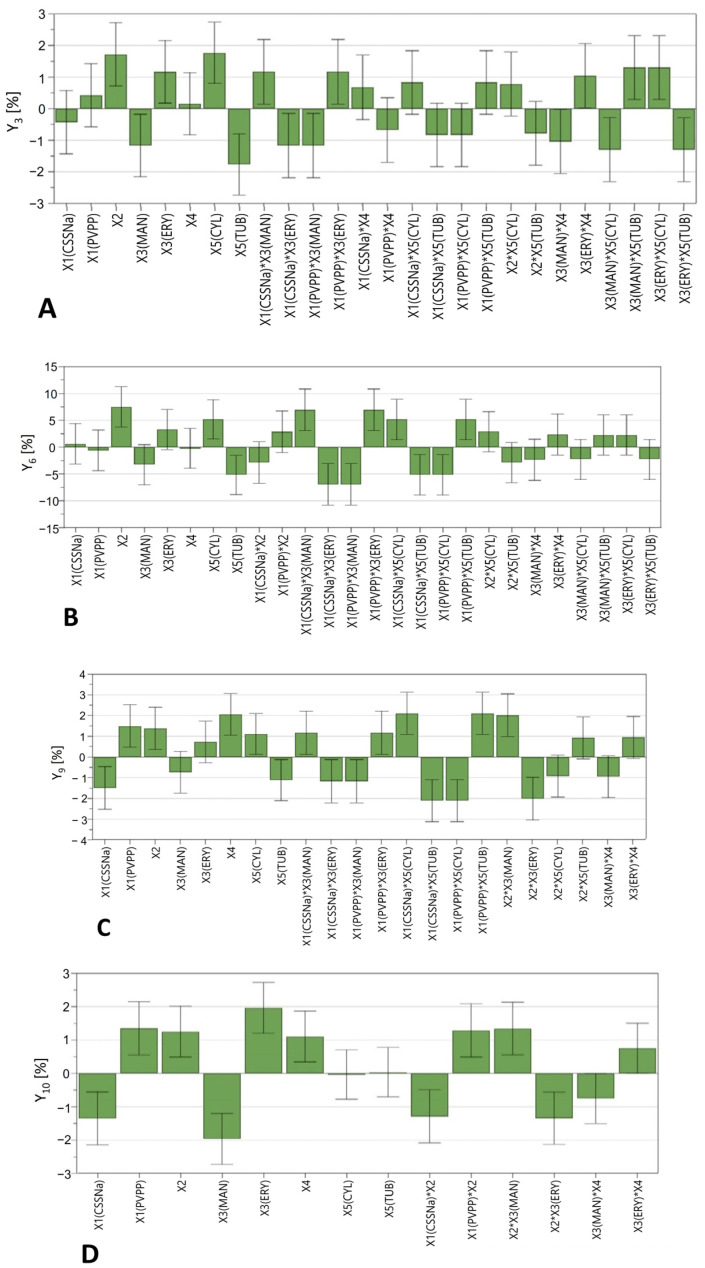
Coefficient plots highlighting the effect of X_1_: type of superdisintegrant (CSSNa, croscarmellose sodium; PVPP, crospovidone); X_2_: plasticiser concentration (% *w*/*w*); X_3_: type of plasticiser (MAN, mannitol; ERY, erythritol); X_4_: layer height (mm); and X_5_: tablets’ geometry on the responses Y_3_: % DS release at 15 min (**A**); Y_6_: % DS release at 60 min (**B**); Y_9_: % DS release at 120 min (**C**); and Y_10_: % DS release at 240 min (**D**).

The effect of the investigated factors on each considered response was interpreted through regression coefficient plots, based on the magnitude and direction of each model term. As shown in Figure 6, the coefficient plot indicates that the type of superdisintegrant (X_1_), plasticiser ratio (X_2_), plasticiser type (X_3_) and tablet geometry (X_5_) significantly affected diclofenac release rate within the first 4 h of dissolution. Furthermore, the presence of a superdisintegrant proved to be insignificant during the first hour (Y_3_–Y_6_) while its intensity increased substantially after 2 and 4 h of dissolution (Y_9_ and Y_10_). Interestingly, different dissolution profiles were observed for tablets containing PVPP compared to those with CSSNa. Specifically, the use of PVPP led to an increase in the drug release rate, whereas it decreased for tablets containing CSSNa (Figure 6C,D). This is consistent with a previous study showing that CSSNa can indeed be less effective in enhancing the drug release from PVA-based printed tablets within a four-hour dissolution interval when compared to PVPP [38]. This behaviour can be attributed to differences in the disintegration mechanism between CSSNa and PVPP. The effect of the disintegration mechanism of these polymers on the dissolution profile of immediate release 3D printed tablets has also been previously investigated and reported [2]. PVPP provides fast swelling and wicking via capillary action and hence fast disintegration due to the volume expansion and the build-up of hydrostatic pressure. In addition, the high crosslink density of the polymer prevents gel formation during its swelling [2]. In contrast, CSSNa is a cellulose-based superdisintegrant which acts by absorbing water and subsequently swelling, resulting in a slower wicking action and hence lower swell rates and tablet disintegration. Additionally, the gelling behaviour of the polymer during its swelling can act as deterrent factor for the developed disintegration forces. Moreover, interactive effects between the type of superdisintegrant and the type of plasticiser were observed. For instance, when CSSNa was combined with MAN, a significant positive effect was observed during the first 60 min of dissolution, while with ERY a considerably slower release was seen. In contrast, tablets containing PVPP and ERY showed a markedly fast release, while the opposite effect appeared in combination with MAN. The influence of the plasticisers was important after 15 min of dissolution (Y_3_) and highly significant at 4 h (Y_9_) of dissolution. Thus, the presence of ERY increased the drug release, whereas the addition of MAN resulted in slower release rates. Another important feature that is highlighted in Figure 6 is that the concentration of plasticisers (X_2_) was significant for the *in vitro* dissolution profile of the printed tablets, with a dominant effect during the first hour of dissolution. Specifically, tablets with a higher concentration of plasticiser (15% *w*/*w*) exhibited faster drug release. This behaviour might be related to the higher moisture content of tablets with a greater amount of plasticiser, leading to faster dissolution.

Another interesting observation is that, although tubular-shaped tablets were designed to enhance the drug release by increasing the contact area between the tablet and the dissolution medium, coefficient plots revealed the opposite effect (Figure 6). The cylindrical-shaped tablets showed a marked increase in DS release, while the tubular design considerably slowed down the dissolution rate. That might be linked to the drug release from water-soluble and swellable polymers such as PVA which is independent of tablet shape and directly related to the relative contribution of drug diffusion and polymer dissolution (surface erosion) [5]. In addition, tablets’ resolution (X_4_) influenced the drug release rate only after 2 h of dissolution. Practically, when tablets were printed at a high resolution (0.05 mm LH), DS release rate was increased compared to tablets with 0.2 mm LH.

Previous studies evidence that the selection of an adequate kinetic model for fitting the drug release data is essential for identifying the drug release mechanism from the printed tablets [23]. Bearing this in mind, the dissolution mechanism for each of the printed dosage forms was identified by applying several mathematical equations and, for each mathematical model, the correlation coefficient (𝑅^2^), Akaike criterion (AIC) and kinetics coefficient (k) were calculated and used as an indicator of the best fitting (Supplementary Table S2).

The results of the analysis evidenced that 25 out of the 30 formulations were best fitted to the Korsmeyer–Peppas model, as they showed the highest regression coefficient and lowest AIC (Supplementary Table S2). However, the N3, N8, N20 and N30 formulations were fitted to the Hixon model, while only N15 fitted best to zero-order kinetics. By fitting the experimental data to the Korsmeyer–Peppas equation, the release coefficient (n) was determined to characterise the kinetics of DS release in the examined formulations. According to the literature, when n < 0.45, drug release is controlled by Fickian diffusion, where the dissolution rate depends on the drug’s diffusion through the matrix, whereas n > 0.89 indicates drug release driven by the polymer’s swelling and erosion. When 0.45 < n < 0.89, drug release is controlled by a non-Fickian diffusion release mechanism involving drug diffusion and swelling simultaneously [23]. Our findings evidenced that for most of the formulations explained by the Korsmeyer–Peppas model, the drug release was an anomalous (non-Fickian) diffusion, since the obtained n values were between 0.45 and 0.89, which is indicative of a diffusion- and erosion-controlled drug release mechanism. However, 10 formulations showed a diffusion exponent greater than 0.89, indicating a case-II relaxation and erosion release mechanism. This behaviour is associated with the swelling and state transitions in hydrophilic glassy polymers, wherein the drug release is primarily governed by polymer relaxation. Based on these findings, it was suggested that the drug release mechanism for cylindrical tablets with high printing resolution consisted of two mechanisms, polymer relaxation (matrix swelling) and drug diffusion. Similar studies have also shown that the drug release of formulations containing water-soluble and swellable polymers such as PVA is dominated by the contribution of polymer dissolution and drug diffusion [5].

## 3. Materials and Methods

### 3.1. Materials

For the preparation of filaments and 3D printed tablets, the following were used: polyvinyl alcohol was used as a water-soluble polymeric matrix, with a degree of hydrolysis of 87–90% and MW of 30.000–70.000, from Sigma-Aldrich (Saint Louis, MO, USA); mannitol (MAN) Parteck^®^ M 200 EMPROVE^®^ from Merck (Darmstadt, Germany); erythritol (ERY) from Sigma-Aldrich (Saint Louis, MO, USA); poloxamer (Lutrol^®^ F68) from BASF (Ludwigshafen, Germany); PEG4000 and PEG1500 from Merck (Darmstadt, Germany); crospovidone (PVPP) Kollidon^®^ CL-F, from BASF (Ludwigshafen, Germany); croscarmellose sodium (CSSNa) Vivasol^®^, from JRS Pharma (Rosenberg, Germany); and diclofenac sodium (DS) from Fagron (Nazareth, Belgium). All the other reagents were of analytical grade purity and were used as supplied.

### 3.2. Preliminary Experiments

Based on the findings of the preliminary studies, a set of eight distinct filament formulations were suggested and extruded utilising the hot melt extrusion (HME) technique. For each formulation, batches of 300 g physical mixtures were prepared, all having the same active substance content (15% *w*/*w* DS) but variable proportions of excipients, as shown in Table 6. To achieve a homogeneous distribution of the raw materials, all formulations were blended for 15 min at 35 rpm in a Turbula T2F shaker mixer (Glen Mills Inc., Clifton, NJ, USA). The physical mixtures were then extruded using a co-rotating, twin-screw extruder (Prism Eurolab 16, Thermo Fisher™ Scientific, Karlsruhe, Germany) with seven electrically heated segments, which could be heated or cooled separately. The extruder was equipped with a DD Flexwall gravimetric feeder (Brabender Technology, Duisburg, Germany), two co-rotating 16 mm twin screws with three mixing zones and one cylindrical die of 1.80 mm. During extrusion, the barrel zone temperatures, screw speed and feed rate were manually controlled through an external data logging system. The extrusion process was operated at constant screw speed (160 rpm) and feed rate (0.3 kg/h). Filaments were collected after 10 min of steady state processing (i.e., constant motor torque) and stored in sealed plastic bags at room temperature to avoid moisture absorption. To link the formulation variables (plasticiser type and ratio) with the energy consumption required at each extrusion and to monitor process fluctuations, the process parameters (barrel temperature, screw speed and feed rate) were kept constant throughout the course of the experiment, while specific mechanical energy (SME) and the torque at screw values were evaluated as dependent parameters.

### 3.3. Filaments’ Characterisation

#### 3.3.1. Drug Content

To determine the DS content of the filaments, 300 mg of each drug-loaded filament, chosen from different spots, was accurately weighed and subsequently dissolved in a 1L volumetric flask containing phosphate buffer of pH 6.8 under magnetic stirring, until complete dissolution. After dissolution, all the samples were filtered through 0.2 µm filters and the DS concentration was determined at 276 nm, using a UV–Vis spectrophotometer (Specord 200 Plus, Analytik Jena, Jena, Germany) [39].

#### 3.3.2. Tensile Measurements

Tensile measurements were performed to evaluate the deformation and mechanical properties of the filaments under tension. Elongation experiments were performed on extruded filaments with a length of 7 cm using a TA.XT Plus Texture Analyser (Stable Micro Systems, Godalming, UK) equipped with a 50 kg load cell and a TA-243 self-tightening roller grip system. The initial distance of separation, test speed and maximum elongation distance were set at 20 mm, 3 mm/s and 140 mm, respectively. The obtained raw data were further converted into stress–strain curves to determine the ultimate tensile stress and ultimate tensile strain (strain of failure) by determining the maximum reached stress and strain, respectively [8]. Modulus of toughness was also calculated by measuring the area under the stress–strain curve, using the trapezoid rule. Exponent software version 6.1.5.0 (Stable Micro Systems, UK) was used for data collection and analysis.

(1) $$\mathrm{Tensile}\;\mathrm{stress}\;(\sigma )=\frac{\mathrm{force}\;}{\mathrm{area}\;\mathrm{of}\;\mathrm{the}\;\mathrm{original}\;\mathrm{cross}-\sec\mathrm{tion}\;}=\frac{F}{\mathrm{Ao}}\;[\mathrm{MPa}]$$

(2) $$\mathrm{Tensile}\;\mathrm{strain}\;(\varepsilon )=\frac{\mathrm{elongation}\;}{\mathrm{original}\;\mathrm{gauge}\;\mathrm{length}\;}=\frac{L-L_{0}}{\mathrm{Lo}}=\mathrm{percent}\;\mathrm{elongation}\;[\%]$$

(3) $$\mathrm{Modulus}\;\mathrm{of}\;\mathrm{toughness}=\int_{a}^{b}F\left(x\right)\mathrm{dx}\;\approx \left(b-a\right)\frac{f\left(a\right)+f\left(b\right)}{2}$$

where σ is the stress, F is the applied force, A is the cross-sectional area, ε is the strain, L is the elongation per unit length and L_0_ is theoretical gauge length.

#### 3.3.3. DSC Measurements

The thermal behaviour of the starting materials, physical mixtures and filaments was evaluated using differential scanning calorimetry (DSC). The measurements were carried out using DSC Q2000 equipment (TA Instruments, Leatherhead, UK) linked with a refrigerated cooling system. Samples were accurately weighed (approximately 5 mg) and then placed in Tzero aluminium pans (TA instruments, Zellik, Belgium). The thermal characteristics (T_g_ and melting enthalpy) were determined via a heat/cool/heat run (cycling scanning) procedure from 20 to 220 °C with a heating/cooling rate of 10 °C/min. After the first heating cycle, the samples were quench cooled at −30 °C, kept at that temperature for 3 min and then heated again up to 220 °C. The first heating cycle was used to determine the melting enthalpy (in the total heat flow signal) and the inflection point of melting endotherm (T_m_), while the second heating cycle was used to calculate the glass transition temperature (T_g_) from the mid-point of the step change in heat flow during the second heating run.

#### 3.3.4. XRD Measurements

The crystallinity of DS and excipients in the drug-loaded filaments was assessed through X-ray powder diffraction (XRD) using a D8 Advance (Bruker, Karlsruhe, Germany) X-ray diffractometer. The XRD patterns were recorded with an ultrafast LynxEye detector. The measurements were performed in the reflection mode using the Bragg–Brentano geometry. The diffractometer was equipped with a curved germanium incident-beam monochromator, to increase resolution by using a monochromatic beam corresponding to Cu-Kα1 radiation (λ = 1.54056 Å). Powder mixtures and filaments were scanned from a 2θ range of 3° to 53°using a step scan mode 0.01°and a scan speed at 0.5 s/step.

#### 3.3.5. Humidity Measurements

The relative humidity of the extruded drug-loaded filaments before printing was determined using a moisture balance. After balance calibration, the samples were weighed in aluminium pans (ø 90 mm, h 8 mm) and heated simultaneously until the unbound moisture evaporated. The loss on drying (LOD) was calculated using the following formula:

(4) $$\mathrm{LOD}\;(\%)=\frac{\mathrm{initial}\;\mathrm{mass}\;\mathrm{of}\;\mathrm{sample}\;–\;\mathrm{mass}\;\mathrm{of}\;\mathrm{sample}\;\mathrm{after}\;\mathrm{drying}\;}{\mathrm{initial}\;\mathrm{mass}\;\mathrm{of}\;\mathrm{sample}}\;\times \;100$$

#### 3.3.6. SME Measurements

The energy consumed by the melting compounds during the hot melt extrusion process, showing the amount of work input from the drive motor into the extruded compound, was expressed as specific mechanical energy (SME) and was calculated using the following formula:

(5) $$\mathrm{SME}={\;n}_{\mathrm{GB}}\frac{2\cdot \pi \cdot n\cdot M}{m\cdot 1000\cdot 60}\;\times \;100\;[\mathrm{KWh}/\mathrm{kg}]$$

where: n_GB_ is the gear box efficiency (the typical value is 0.95), n is the screw speed in rpm, M is the total torque expressed in N and m is the throughput (Kg/h).The calculated SME was used to characterise the extrusion process and link the screw performance with the formulation properties [40].

### 3.4. Development of 3D Printed Tablets Using the Quality by Design Approach

A quality by design approach was followed for the development of 3D printed tablets with DS, as recommended by the International Conference on Harmonization (ICH) guideline for pharmaceutical development Q8 (R2) [41]. Thus, the first step was to establish a QTPP for the printed tablets, summarising the quality criteria that the product must fulfil. Based on the QTPP, which included physical, chemical, biological and microbiological characteristics of the product, the critical quality attributes (CQAs) were established as characteristics which have to be within restricted ranges, limits or distribution in order to have a product of suitable quality.

The potential risk related to the active ingredient, excipients and various unit operations that could further affect the CQAs of the intermediate as well as of the final product was assessed through risk management tools. Specifically, all the potential risk factors were first listed using an Ishikawa diagram. Subsequently, a failure mode effect analysis (FMEA) was implemented to evaluate and prioritise the level of risk associated with each potential critical factor. Each factor was evaluated from the perspectives of potential failure mode effects, potential causes and control methods. Then, to prioritise each factor a risk priority number (RPN) was calculated by multiplying score values ranging from 1 (low impact) to 5 (high impact) assigned to the potential severity (S), occurrence (O) and detection (D) of the failure modes.

Based on the results of risk analysis, three formulation and two process variables were considered as the most critical for their impact on the quality of the printed tablets and were evaluated through DoE methodology. The formulation factors were categorised as two qualitative factors, i.e., the type of superdisintegrant (X_1_) and the type of plasticiser (X_3_), and one quantitative factor, i.e., the percentage of plasticiser (X_2_). Several process variables were identified as critical as well, namely the thickness of the layer (X_4_) and the geometry of the tablet (X_5_). As responses, diclofenac release ratio at ten sampling times, i.e., 5, 10, 15, 30, 45, 60, 75, 90, 120, 240 min (Y_1_ to Y_10_), were chosen. A 2-level full-factorial design with 32 experiments was developed using Modde v.12.1 software (Sartorius Stedim Data Analytics AB), to evaluate the effect of formulation and technological factors on the proposed responses, as summarised in Table 7. The obtained data were fitted through partial least square (PLS) model.

To investigate the impact of geometry on the drug release kinetics, cylindrical and tubular-shaped tablets were designed using 3dsMax software (Autodesk Inc., San Rafael, CA, USA) with the same ratios of length, width and height. The dimensions of standard cylindrical-shaped tablets were the following: 10 mm diameter and 5 mm height. The alternative tubular design had a 4 mm middle channel, proposed as a means to accelerate the diclofenac sodium release rate. The height of the deposited layers was 0.05 or 0.2 mmm, according to the experimental design.

Preparation of filaments through HME followed by printing of tablets was performed for each of the 32 formulations in the experimental design. The printing process was carried out using a MakerBot Replicator 2X FDM 3D printer (MakerBot Inc., Brooklyn, NY, USA). The dosage form design was exported as a stereolithography (.slt) file into the 3D printer software MakerBot Desktop v.3.10.1 (MakerBot Inc., Brooklyn, NY, USA). The lowest printing temperature at which smooth flow and proper deposition of the melted filament were observed was considered adequate. The printing temperature applied was 184 ± 2 °C, the platform temperature was set at 25 ± 3 °C, while the infill % was set at 50% and the number of shells at 2, for all the experiments. Regarding the printing speed, it varied as follows: 20 mm/s, first layer; 20 mm/s, floor surface fills; 40 mm/s, infill printing speed; 40 mm/s, insets; 40 mm/s, outlines; 20 mm/s, roof surface fills; 30 mm/s, sparse roof surface fills.

### 3.5. Tablet Characterisation

#### 3.5.1. Size and Mass Measurements

Immediately after printing, the dimensions of the tablets (diameter and thick-ness/height) were measured using a digital caliper (Bodson, Luik, Belgium) on 10 randomly selected units of each formulation. Weight uniformity was evaluated according to European Pharmacopeia recommendations. Thus, 20 units of each formulation were weighed using an analytical balance (Kern EG, Balingen, Germany), and average mass and uniformity of mass were calculated.

#### 3.5.2. *In Vitro* Dissolution Studies

For all the printed tablets, *in vitro* dissolution experiments were performed using a VK 7010 dissolution system (VanKel Industries, New Jersey, USA) with paddle configuration. The paddle speed and bath temperature were set at 100 rpm and 37 ± 0.5 °C, respectively. The dissolution study was conducted at pH 6.8 (phosphate buffer). Samples were withdrawn at predetermined time intervals of 5, 10, 15, 30, 45, 60, 75, 90, 120 and 240 min and were subsequently filtered and analysed spectrophotometrically (UV-1650PC, Shimadzu Benelux, Antwerp, Belgium) at a 276 nm wavelength, to quantify the released DS. A dissolution test was performed in triplicate for each formulation and cumulative drug release was calculated for each sampling point and expressed as mean and standard deviation.

The evaluation of the DS release profile of each formulation was conducted by fitting the data with six mathematical models for the release kinetics, including zero-order [42], first-order [43], Higuchi [44], Hixson–Crowell [23], Korsmeyer–Peppas [45] and Baker–Lonsdale [46]. All the proposed mathematical models were developed and applied using SigmaPlot v.11.0 software (Systat Software Inc.). The Akaike information criterion (AIC) was applied to test the pertinence of the release models employed. A good model is the one that has the minimum AIC among all the models [47]. Moreover, the coefficient of determination (R^2^) value was used to further examine the goodness-of-fit of each model. Finally, the optimal model was selected based on the highest R^2^ and minimum AIC values.

## 4. Conclusions

This study investigated the release of DS from 3D printed tablets, developed with custom-made drug-loaded filaments. First, filaments developed as intermediate products attained higher elasticity and a smoother surface when ERY was used as a plasticiser, compared to filaments plasticised using MAN. Concerning the 3D printed tablets, they ensured almost 90% of DS was released after 90 min at pH 6.8, regardless of their composition, and the release kinetics was best fitted with the Korsmeyer–Peppas model. Tablets of the same formulation printed with the same geometry at high resolution dissolved slightly faster than those with standard resolution. Moreover, the novel tube design, which was suggested to increase the drug release rate, did not provide better dissolution. On the contrary, this novel design revealed a significant negative effect on dissolution percentage, while tablets with a cylindrical design showed a marked increase in the diclofenac release rate. Finally, the present work demonstrates the potential to combine HME and FDM 3D printing techniques to produce various tailor-made drug delivery systems with desired release kinetics.

## Figures and Tables

**Table 1:** Overview of the formulations investigated in the preliminary studies, the HME process parameters and the appearance of the extruded filaments.

**Preliminary Studies**									
**Exp**	**Polymer**	**% *w*/*w***	**API**	**% *w*/*w***	**Plasticiser**	**% *w*/*w***	**Disintegrant**	**% *w*/*w***	**Feed Rate (g/h)**
PF	PVA	100	-	-	-	-	-	-	300
PF0	PVA	80	DS	20	-	-	-	-	500
PF1	PVA	75	DS	15	Mannitol	10	-	-	300
PF2	PVA	75	DS	15	PEG4000	10	-	-	300
PF3	PVA	89	DS	-	PEG1500	8	PVPP	3	300
PF4	PVA	82	DS	-	PEG1500	15	PVPP	3	300
PF5	PVA	85	DS	-	Lutrol F68	12	PVPP	3	300
PF6	PVA	87	DS	-	Maltodextrin	10	PVPP	3	300
PF7	PVA	87	DS	-	Isomalt	10	PVPP	3	300
PF8	PVA	87	DS	-	Erythritol	10	PVPP	3	300
**HME Process Parameters**									
**Exp**	**Zone 2 (** ** °C ** **)**	**Zone 3 (** ** °C ** **)**	**Zones 4–7 (** ** °C ** **)**	**Screw Speed (rpm)**	**Torque (%)**		**Filament Aspect**		
PF	30	170	190	90	-		Transparent		
	25	160	180	100	-		Transparent		
	20	150	170	110	-		Transparent		
PF0	30	140	160	70	70		Transparent		
	30	130	150	90	40		Transparent		
	30	120	140	95	50		Transparent		
PF1	25	160	180	160	42		Yellow, transparent		
PF2	25	160	180	160	19		White, opacified		
PF3	25	160	180	90	14		White, opacified		
PF4	25	160	180	90	14		White, opacified		
PF5	25	160	180	90	14		White, opacified,		
PF6	25	160	180	90	14		White, opacified		
PF7	25	160	180	90	54		Orange, transparent		
PF8	25	160	180	90	54		White, transparent		

PF: Preliminary filament.

**Table 2:** Overview of the QTPP and CQAs of the diclofenac 3D printed tablets.

QTPP Elements		Target	Is This a CQA?	Justification
Dosage form		Uncoated tablets	No	Pharmaceutical equivalence requirement: same dosage form.
Dosage design		Cylindrical/tubular tablets	No	Pharmaceutical equivalence requirement: same dosage form. The geometrical design may affect the drug release performance and thus product efficacy and patient compliance.
Route of administration		Oral	No	Pharmaceutical equivalence requirement: same route of administration. Ensures patient compliance and acceptability.
Dosage strength		50 mg	Yes	To ensure therapeutic efficacy.
Biopharmaceutical properties		T_max_ not greater than 2 h	Yes	Needed to ensure rapid onset and efficacy.
Product quality attributes	Identification	Positive for diclofenac Na	No	API identification is critical for safety and efficacy. However, this feature will be monitored at drug product release.
	Assay of active substances	90% to 110% of the labelled content	Yes	Drug assay and mass variability are directly linked with the drug content uniformity and affect safety and efficacy.
	Average mass Uniformity of mass	The unit dose variation should not exceed ±5% of the average mass	Yes	Drug assay and mass variability are directly linked with the drug content uniformity and affect safety and efficacy.
	Dissolution at pH 6.8	Not less than 20% at 15 min Not less than 50% at 60 min Not less than 95% at 120 min	Yes	Failure to meet dissolution specification can impact bioavailability. Both formulation and process variables affect dissolution profile.

**Table 3:** Physicochemical characteristics of filaments. Data are expressed as mean ± SD (n = 3).

Filaments	Drug Content (%)	Loss on Drying (%)	Torque (Nm)	SME (Kwh/Kg)	Ultimate Tensile Strength (MPa)	Strain Failure (%)	Toughness (Jm^−1^)
F	-	-	-	-	67.27 ± 9.74	19.98 ± 1.00	4.9 ± 0.7
F1	93.78	1.80	9.8	0.52	54.83 ± 5.60	17.38 ± 0.81	3.3 ± 0.7
F2	96.22	1.81	10	0.53	53.64 ± 1.62	17.44 ± 0.87	3.3 ± 0.4
F3	95.63	1.90	7.9	0.42	40.50 ± 3.30	14.28 ± 0.40	2.3 ± 0.3
F4	98.05	2.67	8.8	0.47	32.58 ± 6.18	14.43 ± 1.02	2.0 ± 0.5
F5	94.90	1.30	9.3	0.49	38.87 ± 4.20	21.02 ± 1.64	3.6 ± 1.1
F6	93.44	1.41	9.6	0.51	43.43 ± 1.91	23.67 ± 3.02	3.6 ± 0.3
F7	94.45	1.39	8.4	0.45	25.47 ± 1.71	449.6 ± 80.8	99.6 ± 22.9
F8	97.24	2.10	8.8	0.47	29.86 ± 0.49	433.0 ± 375.0	162.4 ± 4.6
PVA *	-	-	-	-	56.96 ± 1.94	34.06 ± 2.73	11.2 ± 0.3

* PVA: PVA commercial filament.

**Table 4:** The composition and physical characteristics of 3D printed tablets.

Exp.	X_1_	X_2_	X_3_	LH (X_4_)/Design (X_5_)	Mass (mg)	CV %	Exp.	X_1_	X_2_	X_3_	LH (X_4_)/Design (X_5_)	Weight (mg)	CV %
N1	CSSNa	10	MAN	0.2/CYL	380.5	0.10	N17	CSSNa	10	ERY	0.2/CYL	450.1	0.10
N2	CSSNa	10	MAN	0.05/CYL	381.4	0.10	N18	CSSNa	10	ERY	0.05/CYL	449.2	0.10
N3	CSSNa	10	MAN	0.2/TUB	310.7	0.01	N19	CSSNa	10	ERY	0.2/TUB	374.2	0.10
N4	CSSNa	10	MAN	0.05/TUB	356.7	0.20	N20	CSSNa	10	ERY	0.05/TUB	393.6	0.10
N5	PVPP	10	MAN	0.2/CYL	424.4	0.10	N21	PVPP	10	ERY	0.2/CYL	339.9	0.10
N6	PVPP	10	MAN	0.05/CYL	432.6	0.10	N22	PVPP	10	ERY	0.05/CYL	429.1	0.01
N7	PVPP	10	MAN	0.2/TUB	315.6	0.10	N23	PVPP	10	ERY	0.2/TUB	365.5	0.10
N8	PVPP	10	MAN	0.05/TUB	380.0	0.10	N24	PVPP	10	ERY	0.05/TUB	333.3	0.01
N9	CSSNa	15	MAN	0.2/CYL	395.8	0.10	N25	CSSNa	15	ERY	0.2/CYL	399.6	0.10
N10	CSSNa	15	MAN	0.05/CYL	409.4	0.01	N26	CSSNa	15	ERY	0.05/CYL	342.3	0.01
N11	CSSNa	15	MAN	0.2/TUB	326.0	0.20	N27	CSSNa	15	ERY	0.2/TUB	334.0	0.10
N12	CSSNa	15	MAN	0.05/TUB	361.6	0.01	N28	CSSNa	15	ERY	0.05/TUB	299.0	0.10
N13	PVPP	15	MAN	0.2/CYL	428.6	0.01	N29	PVPP	15	ERY	0.2/CYL	418.4	0.10
N14	PVPP	15	MAN	0.05/CYL	373.2	0.10	N30	PVPP	15	ERY	0.05/CYL	394.6	0.10
N15	PVPP	15	MAN	0.2/TUB	348.7	0.10	N31	PVPP	15	ERY	0.2/TUB	312.3	0.10
N16	PVPP	15	MAN	0.05/TUB	369.2	0.10	N32	PVPP	15	ERY	0.05/TUB	385.7	0.01

X_1_: type of superdisintegrant (CSSNa, croscarmellose sodium; PVPP, crospovidone); X_2_: plasticiser concentration (%, *w*/*w*); X_3_: type of plasticiser (MAN, mannitol; ERY, erythritol); X_4_: layer height (mm); X_5_: tablet geometry (CYL, cylindrical design; TUB, tubular design); CV: coefficient of variation (%).

**Table 5:** Overview of the experimental results of the full factorial design.

Exp Name	Y_1_	Y_2_	Y_3_	Y_4_	Y_5_	Y_6_	Y_7_	Y_8_	Y_9_	Y_10_
N1	8.51	15.51	19.38	45.59	70.92	89.49	95.05	94.68	95.02	94.30
N3	7.11	11.81	17.34	33.63	48.42	62.78	76.50	87.06	92.21	92.98
N5	7.18	13.22	19.52	37.36	53.30	67.57	81.58	93.91	98.32	97.53
N6	6.06	11.25	15.26	26.84	37.40	48.00	60.62	75.60	93.37	95.03
N7	8.68	15.49	22.09	41.53	59.33	75.22	85.55	92.44	97.11	96.72
N8	4.88	8.87	12.94	26.46	37.93	50.03	62.99	73.36	88.58	92.67
N9	7.95	15.14	22.28	41.67	59.39	75.49	89.08	92.52	92.14	93.36
N10	8.09	15.15	22.45	42.86	60.78	85.72	95.33	95.70	99.77	96.05
N11	7.49	14.35	20.64	39.42	56.07	75.71	88.66	94.84	96.34	96.66
N12	8.03	12.09	16.79	32.60	46.92	61.30	73.41	84.40	93.91	97.07
N13	8.32	15.72	23.52	42.31	57.89	74.34	88.28	98.18	101.84	102.55
N14	7.41	13.87	21.30	42.23	61.69	83.29	95.38	95.71	96.69	96.58
N15	7.15	13.25	19.15	36.11	51.90	67.66	81.10	91.61	99.80	100.91
N16	8.78	14.92	21.07	39.46	57.18	72.94	87.53	97.52	102.38	102.91
N17	15.57	14.20	20.20	36.54	51.77	66.47	78.95	89.77	99.59	101.89
N18	7.22	13.43	19.39	36.36	51.76	66.15	81.60	95.56	98.36	98.79
N19	7.07	10.51	14.77	27.64	39.66	51.14	62.00	74.87	90.27	102.70
N20	4.31	7.94	11.82	23.02	34.54	45.94	56.84	67.15	85.30	95.83
N21	11.48	22.47	31.41	52.13	70.99	80.55	93.21	99.25	100.46	101.38
N22	5.85	11.14	16.30	33.75	49.02	62.48	81.26	93.31	96.75	99.62
N23	9.08	13.96	19.60	34.96	49.83	64.27	77.88	90.49	104.73	108.76
N24	7.22	12.67	18.17	35.71	54.29	71.97	83.19	88.95	97.10	96.81
N25	24.22	24.90	28.19	49.99	74.44	96.73	99.26	100.49	99.99	101.19
N26	8.24	15.16	22.05	39.93	61.10	78.47	85.00	89.67	92.78	92.18
N27	6.39	11.19	17.53	26.90	37.74	48.33	59.97	71.48	94.42	98.64
N28	37.96	35.87	43.18	73.71	89.40	95.64	95.81	96.08	97.05	97.61
N29	9.60	17.78	25.51	46.26	69.24	92.13	102.30	101.49	101.06	104.56
N30	9.22	17.90	27.01	59.66	85.79	89.84	91.94	92.38	90.10	90.02
N31	15.61	27.72	39.79	70.35	92.34	98.12	102.60	102.01	102.48	101.55
N32	7.06	13.17	18.67	34.21	47.76	59.55	72.41	79.00	94.97	100.16

Y_1_ to Y_10_, % DS released at 5, 10, 15, 30, 45, 60, 75, 90, 120 and 240 min, in pH 6.8.

**Table 6:** Overview of filaments’ composition prepared through HME.

Filament	Active Substance		Polymer Matrix		Plasticiser		Disintegrant	
	Type	%	Type	%	Type	%	Type	%
F1	DS	15	PVA	72	MAN	10	CSSNa	3
F2	DS	15	PVA	72	MAN	10	PVPP	3
F3	DS	15	PVA	67	MAN	15	CSSNa	3
F4	DS	15	PVA	67	MAN	15	PVPP	3
F5	DS	15	PVA	72	ERY	10	CSSNa	3
F6	DS	15	PVA	72	ERY	10	PVPP	3
F7	DS	15	PVA	67	ERY	15	CSSNa	3
F8	DS	15	PVA	67	ERY	15	PVPP	3

**Table 7:** The studied variables and their levels of variation and the evaluated responses.

	Factors	Design Level		Responses			
		−1	+1				
X_1_	Superdisintegrant type [-]	CSSNa	PVPP	Y_1_	% DS release at 5 min	Y_6_	% DS release at 60 min
X_2_	Plasticiser conc. [%, *w*/*w*]	10	15	Y_2_	% DS release at 10 min	Y_7_	% DS release at 75 min
X_3_	Plasticiser type [-]	MAN	ERY	Y_3_	% DS release at 15 min	Y_8_	% DS release at 90 min
X_4_	Layer height [mm]	0.05	0.2	Y_4_	% DS release at 30 min	Y_9_	% DS release at 120 min
X_5_	Tablet geometry [-]	CYL	TUB	Y_5_	% DS release at 45 min	Y_10_	% DS release at 240 min

CSSNa, croscarmellose sodium; PVPP, crospovidone; MAN, mannitol; ERY, erythritol; CYL, cylindrical design; TUB, tubular design.

## Data Availability

Data is contained within the article and Supplementary Material.

## Supplementary Materials

The following supporting information can be downloaded at: https://www.mdpi.com/article/10.3390/ph16081062/s1, Table S1. Overview of the failure mode effect analysis with the amount of risk of each factor; Figure S1. In-vitro dissolution profiles of diclofenac sodium 3D printed tablets with 10% *w*/*w* mannitol (A); 15% *w*/*w* mannitol (B); 10% *w*/*w* erythritol (C) and 15% *w*/*w* erythritol (D) with varied type of super disintegrant, geometry and percent of infill; Table S2. Overview of drug release modelling parameters for Baker-Lonsdale, Peppas and Korsmeyer and Hixon and Crowell models; Table S3. Overview of drug release modelling parameters for Higuchi, First-order and Zero-order models.
