# Supplementary material for: Development of Diclofenac Sodium 3D Printed Cylindrical and Tubular-Shaped Tablets through Hot Melt Extrusion and Fused Deposition Modelling Techniques
Source: Pharmaceuticals (Basel). 2023 Jul 26;16(8):1062. doi: 10.3390/ph16081062 (PMC10459775; doi:10.3390/ph16081062)
Supplement: Supplementary file 1 [file pharmaceuticals-16-01062-s001.zip › pharmaceuticals-2494394-supplementary.pdf]

pharmaceuticals

Supplementary Materials: Development of diclofenac sodium 3D printed cylindrical and tubular-shaped tablets through hot melt extrusion and fused deposition modelling techniques

Tryfon Digkas, Alina Porfire, Jeroen Van Renterghem, Aseel Samaro, Gheorghe Borodi, Chris Vervaet, Andrea Gabriela Crișan, Sonia Iurian, Thomas De Beer, Ioan Tomuta

Table S1. Overview of the failure mode effect analysis with the amount of risk of each factor

Operation	CMAs/CPPs	Potential Failure mode	Potential Failure effect	Potential causes	O	S	D	RPN	Control Methods
Formulation	Excipients properties	Drug-excipients incompatibility<br>Viscosity matching<br>Limited interdiffusion	Inadequate filaments for FDM printing<br>Swelling of the die	Low/high Tg; non-thermoplastic polymers; drug-excipients interaction; polymer viscoelastic properties<br>Inadequate extrusion temperature	04	04	03	48	Pre-formulation studies
	Blends melt viscosity	Low melt viscosity	Liquid solid dispersions<br>Inadequate filaments for FDM	Higher extrusion temperature	04	04	02	48	Pre-formulation studies
		High melt viscosity	Extrusion failure due to high torque at screws	Lower extrusion temperature	05	04	03	60	Preformulation studies; Addition of plasticizer
HME Process	Temperature profile	Incongruent melting<br>Inadequate amorphization of API	Assay content<br>Tablet dissolution – inadequate drug release<br>Extrusion operation stop	Low temperature<br>- low melt viscosity<br>- Maximum torque	04	05	03	60	Preformulation studies
		Drug/excipients decomposition	Inadequate filaments for FDM printing	High temperature					
	Screw speed	Drug content uniformity	Assay content<br>Extrusion operation stop	Low screw speed<br>Maximum torque	03	05	02	30	Preformulation studies
		Drug/excipients degradation	Assay content<br>Tablets dissolution - inadequate drug release	High screw speed<br>Elevated temperature of the melted compound					
		Incongruent melting<br>Limited intermolecular contact between API/Polymer		High screw speed<br>Short residence times					
	Feed-rate	Drug/excipients degradation<br>Wrinkled extrudates	Assay content	Low throughput → long residence time<br>High throughput → short residence time	03	03	02	18	Preformulation studies
	Die geometry	Appropriate selection of HME die	Inadequate filaments dimension<br>FDM printing failure	Inappropriate use of HME die	03	04	03	48	Preformulation studies
	Screw configurations	Appropriate selection of screws configuration	Inadequate mixing of melt compound<br>Lack of dosage uniformity	Inappropriate selection of screw configuration	03	03	03	27	Literature research
	Physico-mecanical and rheological	Amorphous/crystalline state of API & excipients	Assay content<br>Drug release rate	Low extrusion temperature<br>High throughput →Short residence time	04	04	03	32	DSC and XRD analysis on both physical mixtures

	properties of the filaments								and drug-loaded filaments
		Inconsistent diameter of filaments	Variability in the deposed quantity → weigh variability →Assay content	Inconsistent extrusion force	04	04	03	32	Throughput, screw speed, temperature standardized. A pull roller device with constant speed
		Stiffness & strength of filament	Fragmentation, bend, break, slip during FDM feeding of filaments	Inadequate quantitative and qualitative formulation	05	04	03	60	Two plasticizers were added. Their plasticization effect was investigated via DoE
		Moisture content	FDM printing complications/failure Filaments stability → Drug Assay	Moisture sorption	04	03	02	24	Filaments stored at sealed plastic bags at room temperature
FDM 3D printing	Printing temperature	Filaments degradation Clogged nozzle	FDM printing failure	High printing temperature Low printing temperature	05	05	04	100	Preliminary studies
	Percent of infill	Reduced/enhanced internal porosity/surface area of tablets	Drug release rate	Low/high infill percentage	04	04	02	32	Preliminary studies
	Layer Height	Defects on tablet geometry and surface area	Drug release rate	Low layer height (printing resolution)	04	04	03	48	DoE for process understanding
	Printing Speed	Filament degradation Defects on tablet geometry and surface area	FDM printing failure Drug release rate	Low printing speed High residence time High printing speed	03	04	02	24	Preliminary printing tests
	Equipment limitations	Filament degradation	Defects on tablets geometry and surface area	Lack of cooling system on printer building plate	05	04	03	60	Addition of Acid citric / 10′ process pause every 10 prints
Environment	Relative humidity	Stability of drug-loaded filaments and 3D printed tablets	Recrystallisation Drug release rate	High moisture sorption	03	03	02	18	Filaments and printed tablets stored at sealed plastic bags

Abbreviations: API: Active pharmaceutical ingredient, O: Occurrence, S: Severity, D: Detectability, RPN: Risk priority number.

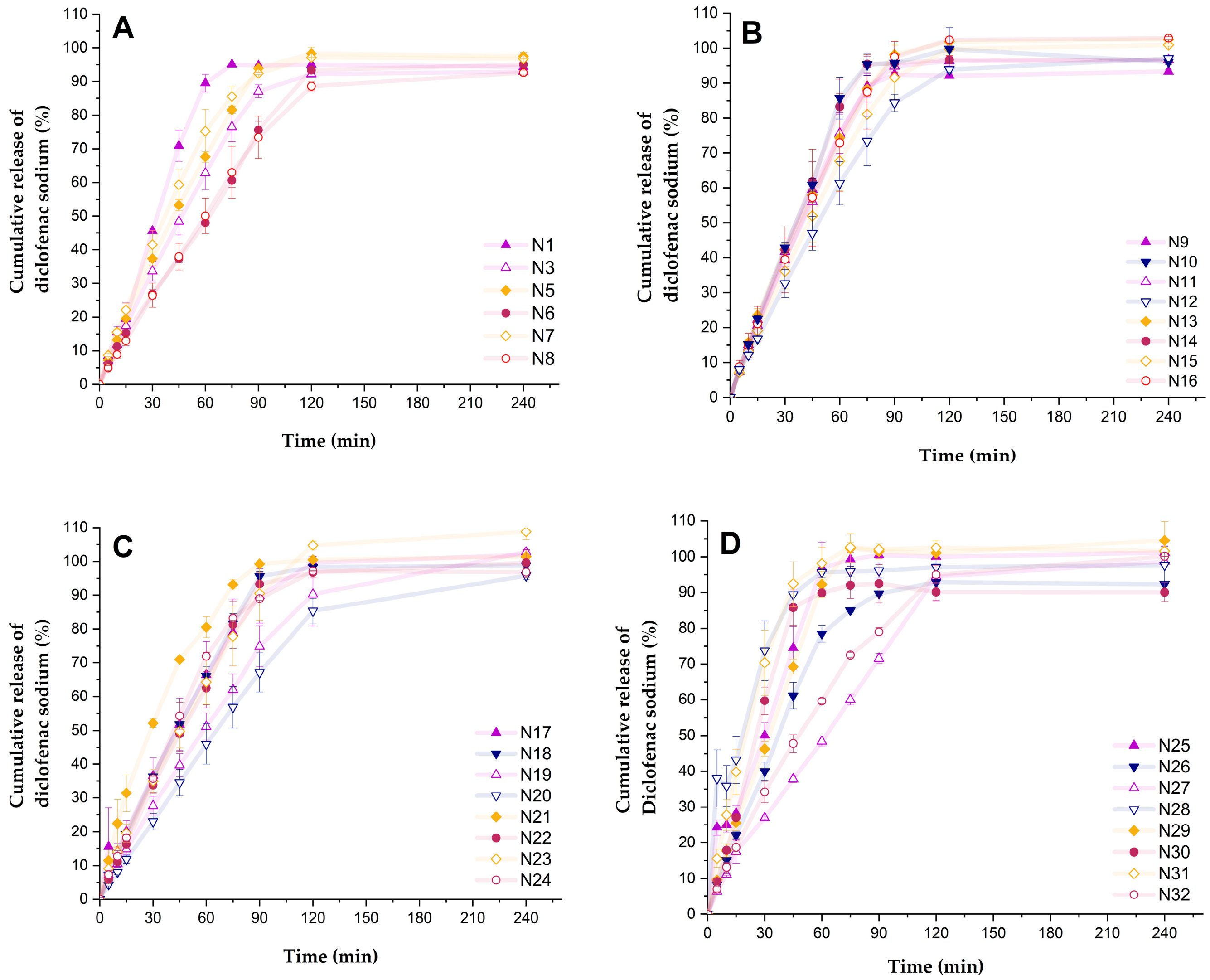

Figure S1. In-vitro dissolution profiles of diclofenac sodium 3D printed tablets with 10% w/w mannitol (A); 15% w/w mannitol (B); 10% w/w erythritol (C) and 15% w/w erythritol (D) with varied type of super disintegrant, geometry and percent of infill.

Table S2. Overview of drug release modelling parameters for Baker-Lonsdale, Peppas and Korsmeyer and Hixon and Crowell models.

	Baker and Lonsdale			Peppas and Korsmeyer				Hixon and Crowell		
	R^2	AIC	K_0	R^2	AIC	K_P	n	R^2	AIC	K_h
N1	0.891	45.560	0.002	0.998	22.410	1.468	1.008	0.984	34.230	0.007
N2										
N3	0.933	67.210	0.002	0.991	51.020	2.756	0.752	0.997	40.67	0.005
N4										
N5	0.923	59.880	0.002	1.000	17.240	1.968	0.862	0.994	39.15	0.005
N6	0.913	60.25	0.001	1.000	10.79	1.372	0.933	0.991	42.61	0.005
N7	0.927	51.72	0.002	0.998	29.65	2.892	0.813	0.996	31.18	0.007
N8	0.938	74.390	0.001	0.958	72.500	4.784	0.568	0.994	51.590	0.004
N9	0.924	51.070	0.002	1.000	16.130	2.222	0.858	0.996	30.430	0.006
N10	0.909	53.590	0.002	0.998	29.830	1.974	0.906	0.988	39.460	0.007
N11	0.922	60.820	0.002	0.997	37.020	2.335	0.835	0.994	41.000	0.006
N12	0.926	59.366	0.002	0.999	27.424	2.067	0.847	0.996	36.774	0.005
N13	0.930	59.590	0.002	0.999	26.180	2.708	0.803	0.995	38.990	0.006
N14	0.906	53.780	0.002	0.999	25.880	1.808	0.925	0.989	39.220	0.007
N15	0.924	59.590	0.002	0.995	22.370	1.936	0.862	0.995	38.040	0.005
N16	0.912	60.918	0.002	1.000	15.844	1.469	0.927	0.989	44.845	0.005
N17	0.933	58.795	0.002	1.000	22.231	2.553	0.805	0.997	35.247	0.006
N18	0.919	60.380	0.002	1.000	-0.820	1.753	0.889	0.992	42.470	0.005
N19	0.925	66.150	0.001	0.999	27.550	1.565	0.851	0.995	42.040	0.004
N20	0.934	74.690	0.001	0.970	68.890	3.594	0.620	0.995	49.130	0.003
N21	0.939	58.533	0.002	0.998	31.629	3.250	0.760	0.991	43.053	0.006
N22	0.910	62.136	0.002	0.998	32.243	1.714	0.906	0.988	46.479	0.006
N23	0.928	58.430	0.002	1.000	11.120	1.965	0.851	0.986	45.600	0.005
N24	0.908	53.055	0.002	0.998	30.164	1.739	0.924	0.990	37.552	0.006
N25	0.929	42.920	0.003	0.988	34.450	4.112	0.763	0.975	36.690	0.008
N26	0.914	53.455	0.002	0.994	37.229	2.540	0.851	0.990	38.474	0.007
N27	0.918	66.870	0.001	0.999	30.320	1.298	0.892	0.981	53.880	0.004
N28	0.970	30.460	0.004	0.983	29.590	12.345	0.517	0.955	32.400	0.013
N29	0.907	44.560	0.002	1.000	12.840	1.971	0.937	0.988	32.350	0.007
N30	0.909	45.530	0.003	0.987	35.920	3.174	0.837	0.990	37.280	0.009
N31	0.932	35.320	0.003	0.999	14.970	4.681	0.787	0.982	28.890	0.005
N32	0.950	64.461	0.002	0.996	43.570	3.707	0.691	1.000	19.943	0.005

Table S3. Overview of drug release modelling parameters for Higuchi, First-order and Zero-order models.

	Higuchi			First order			Zero order		
	R^2	AIC	K_P	R^2	AIC	K_1	R^2	AIC	K_0
N1	0.923	41.580	9.465	0.971	37.795	0.024	0.998	20.459	1.515
N2									
N3	0.936	64.740	7.707	0.976	58.052	0.016	0.983	55.033	0.891
N4									
N5	0.954	53.930	8.535	0.985	47.031	0.019	0.996	36.996	1.097
N6	0.942	55.04	8.000	0.981	48.47	0.017	0.999	24.28	1.035
N7	0.959	45.89	9.681	0.987	39.78	0.024	0.989	38.71	1.357
N8	0.954	69.480	6.556	0.980	63.501	0.012	0.841	83.280	0.537
N9	0.953	45.840	8.895	0.988	38.359	0.021	0.995	31.885	1.253
N10	0.943	48.430	9.521	0.976	44.507	0.023	0.996	31.951	1.347
N11	0.926	58.450	8.576	0.967	54.265	0.019	0.997	35.456	1.108
N12	0.955	53.423	8.452	0.987	45.607	0.019	0.994	39.551	1.085
N13	0.963	53.040	9.246	0.985	47.711	0.022	0.990	44.491	1.182
N14	0.940	48.740	9.413	0.976	44.442	0.023	0.998	28.251	1.335
N15	0.924	57.590	7.958	0.971	52.154	0.017	0.999	22.521	1.030
N16	0.944	55.453	8.363	0.977	50.371	0.018	0.999	26.790	1.081
N17	0.963	52.156	8.784	0.989	44.938	0.020	0.991	43.227	1.123
N18	0.950	54.600	8.502	0.981	48.889	0.019	0.998	32.621	1.096
N19	0.926	64.030	6.723	0.975	56.358	0.012	0.998	33.275	0.785
N20	0.939	71.970	6.114	0.980	62.969	0.011	0.892	79.366	0.524
N21	0.970	50.870	9.304	0.983	48.390	0.022	0.984	47.757	1.183
N22	0.945	56.361	8.919	0.975	52.081	0.020	0.997	36.036	1.152
N23	0.928	56.470	7.743	0.973	50.657	0.016	1.000	0.000	1.000
N24	0.939	48.259	8.997	0.979	42.887	0.021	0.996	30.774	1.276
N25	0.960	37.620	10.694	0.967	38.445	0.030	0.974	36.996	1.665
N26	0.948	48.065	9.856	0.979	44.002	0.025	0.989	39.420	1.388
N27	0.920	64.620	6.656	0.968	58.589	0.012	0.999	24.610	0.779
N28	0.979	26.290	11.822	0.979	28.686	0.037	0.938	33.911	1.998
N29	0.938	40.190	9.745	0.977	36.412	0.025	0.999	18.164	1.548
N30	0.942	40.970	10.788	0.979	37.105	0.031	0.981	36.471	1.698
N31	0.930	33.480	10.944	0.969	31.591	0.031	0.998	19.071	1.917
N32	0.977	55.627	8.320	0.995	43.154	0.018	0.964	61.435	0.940
